# An integrated model to explore Chinese Gen Z consumers' second-hand clothing purchase intention

**DOI:** 10.3389/fpsyg.2026.1783223

**Published:** 2026-07-10

**Authors:** Qi Zhou, Xiaofen Ji, Liling Cai, Liyuan Zhou

**Affiliations:** 1 School of Art and Design, Zhejiang Sci-Tech University, Hangzhou, China; 2 College of Art, Jiujiang University, Jiujiang, China; 3 International Institute of Fashion Technology, Zhejiang Sci-Tech University, Hangzhou, China; 4 China National Silk Museum, Hangzhou, China; 5 Dougou Central Primary School, Wuhu, China

**Keywords:** Gen Z consumers, integrated model, purchase intention, second-hand clothing, theory of planned behavior

## Abstract

To develop a comprehensive framework for understanding the factors associated with Chinese Generation Z (Gen Z) consumers' intention to purchase second-hand clothing (SHC), this study analyzed a valid sample of 967 Chinese Gen Z respondents obtained through stratified quota sampling. Structural equation modeling (SEM) was conducted using AMOS. Guided by the Theory of Planned Behavior (TPB), the proposed model incorporated additional constructs related to values (biospheric and altruistic values) and social influence (subjective norm, group norm, and social identity). Model comparison using their variance explained (*R*^2^) for purchase intention showed that the integrated model exhibited improved in-sample explanatory performance over the baseline TPB, explaining 53.7% of the variance in purchase intention vs. 34.2% for the TPB alone. Altruistic values (β = 0.154, *p* < 0.01), biospheric values (β = 0.143, *p* < 0.01), attitude (β = 0.168, *p* < 0.001), subjective norm (β = 0.187, *p* < 0.001), and social identity (β = 0.394, *p* < 0.001) were positively and significantly associated with purchase intention. Exploratory multi-group SEM revealed no significant differences by gender or city tier. Given the non-probability sampling and cross-sectional design, all findings should be interpreted as associational rather than causal. This study offers new insights into previously underexplored factors associated with SHC purchase intention among Chinese Gen Z consumers by integrating value-based and social influence constructs with the TPB.

## 1 Introduction

As the world's largest textile and apparel producer, China plays a significant role in the fast fashion consumption sector (Lang et al., 2019). The textile industry contributes substantially to environmental pollution, and its byproducts have been demonstrated to pose considerable risks to ecosystems and public health (Bjurbäck, 2015). Globally, more than 30 million tons of clothing are discarded each year, resulting in considerable resource waste and posing serious urban pollution challenges (Rausch and Kopplin, 2021). Researchers have suggested that shifting from first-hand to second-hand consumption can effectively reduce textile waste and mitigate environmental pollution (Machado et al., 2019). With an increasing number of young consumers embracing sustainable consumption practices, the second-hand clothing (SHC) market has experienced steady growth in recent years (Su et al., 2019).

Against the backdrop of China's rapidly expanding fashion resale market and the growth of the SHC sector, a comprehensive understanding of consumers' SHC purchase intentions has become imperative for industry stakeholders. Such insights are essential to support sustainable development initiatives and maximize their potential benefits. Although recent studies have increasingly emphasized the exploration of consumers' SHC purchase intentions (Kian et al., 2022; Madeline et al., 2023; Nawaz Muhammad Zahid and Meng, 2023), this body of work exhibits two key limitations that the present study aims to address.

First, a contextual and demographic oversight is evident. Existing research has predominantly focused on young consumers in Western, individualistic societies (Madeline et al., 2023; Mazanec and Harantová, 2024) or in other Asian markets (Kian et al., 2022). Given that drivers of sustainable consumption are highly sensitive to cultural and economic contexts (Kim and Kim, 2025; Weder et al., 2025), this focus has resulted in a notable gap. Chinese Generation Z (Gen Z), a cohort projected to become the world's most significant consumer segment by 2030 with unprecedented growth in purchasing power (Kim et al., 2020), remains critically underexamined. This generation demonstrates substantially greater concern for environmental and socio-ecological issues than previous generations (Kim et al., 2020; Nanda, 2020) and shows a strong preference for adopting sustainable products (Masserini et al., 2024). Regarded as opinion leaders in consumption, they play a pivotal role in shaping and propagating emerging consumption trends (Lee, 2023). Furthermore, generational theory posits that values and consumption behaviors are shaped by unique socio-historical contexts, making findings from Western cohorts not directly transferable to their Chinese counterparts (Amarullah et al., 2025; Debevec et al., 2013; Rahulan et al., 2015). Chinese Gen Z are digital natives who have come of age within a unique hybrid culture that blends deeply rooted Confucian collectivism with globalized digital individualism (Tsai and Bagozzi, 2014; Fung, 2011). This distinctive positioning further underscores the limitations of generalizing findings from other populations.

Second, a theoretical insufficiency exists in applying standard behavioral models to this group. The Theory of Planned Behavior (TPB) has been widely used to study SHC purchase intentions, but its standard constructs may not fully capture the nuanced motivations of Chinese Gen Z. As a cohort socialized in an era of severe environmental crises and hyper-connected social media, Chinese Gen Z exhibits consumption decisions that are likely governed by a complex interplay between internalized values and external social influence amplified through digital networks. These factors are underemphasized by the conventional TPB (ElHaffar et al., 2020). Extending the TPB with additional constructs is not inherently novel, as numerous studies have added values, norms, or identity variables to improve its explanatory power in various contexts (Tewari et al., 2022; Bhardwaj et al., 2023). However, the present study differs from prior extensions in three specific ways. First, the present study focuses on a theoretically distinct population—Chinese Gen Z. Given their unique hybrid cultural orientation (Confucian collectivism combined with digital individualism), the patterns of associations observed in this cohort may differ from those found in Western or other Asian samples. Initial, exploratory evidence regarding which factors are correlated with SHC purchase intention within this understudied population is provided by this study. Second, this study contributes by incorporating three forms of social influence within one model into the analysis of Chinese Gen Z, and the relative strengths of their correlations were compared. By this design, a direct comparison of the relative strength of their associations with purchase intention is permitted, a comparison that has not been previously reported for Chinese Gen Z in the SHC context. Third, an explicit comparison of the extended model's in-sample explanatory performance (i.e., variance explained in purchase intention) against a baseline TPB model is provided. Many prior extensions have reported the fit of the extended model without quantifying the additional variance gained over the unextended TPB.

The core factor of consumer choice is value. Claeys et al. (1995) asserted that value serves as the ultimate source of selection criteria underlying purchasing behavior. Recent research has further emphasized the critical role of values in their association with the willingness of consumers to engage in green consumption (Ahmad et al., 2020; Wang et al., 2020). According to Homer and Kahle (1988), values are associated with pro-environmental behavior. Different generations have different values due to the environments they were in during their growth, and these values tend to stabilize basically after reaching adulthood (Schewe and Meredith, 2004). Biospheric and altruistic values represent relatively stable, distal factors. These values are hypothesized to be associated with purchase intention and attitude. Specifically, when consumers internalize biospheric values or altruistic values, they are associated with a more favorable attitude toward SHC. This favorable attitude is positively associated with purchase intention. This value-attitude-behavior hierarchy model (Homer and Kahle, 1988) provides a theoretical basis.

For over two millennia, traditional Chinese values, rooted in Confucian philosophy, have profoundly influenced the cultural values of Chinese society (Fung, 2011). This form of collectivism, cultural and social dynamics often exert a substantial influence on consumer behavior. For example, in collectivist societies like China, interpersonal relationships are more strongly associated with purchasing decisions compared to individualistic societies (Xin et al., 2025). With the progression of globalization and modernization, Chinese Gen Z, often referred to as digital natives, is characterized by the coexistence of both Confucian collectivism and digital individualism (Tsai and Bagozzi, 2014). In contrast to previous generations, Chinese Gen Z actively participates in social media platforms (Tsai and Bagozzi, 2014), where they are frequently exposed to positive information concerning SHC. This includes content related to environmental protection initiatives and the promotion of sustainable fashion practices. Moreover, sustainable behaviors and environmental actions in the digital economy are often performed to gain social recognition (Kim et al., 2021). Therefore, it is essential to integrate multiple forms of social influence into the theoretical framework of this study.

Consequently, this study aims to bridge this dual gap by developing and testing an extended TPB model that integrates biospheric values, altruistic values, and social influence (subjective norm, group norm, social identity) specifically to investigate the associations with SHC purchase intentions of Chinese Gen Z consumers. The specific contributions are as follows. First, an empirical comparison was conducted to determine whether, within the current sample, the extended model explains more variance in purchase intention than the baseline TPB (i.e., in-sample explanatory performance). Second, the relative strength of different social influence constructs (subjective norm, group norm, and social identity), was examined within a single model, thereby providing initial insights into which type of social influence is most strongly correlated with purchase intention for this demographic.

The structure of this paper is organized as follows. The next section presents theoretical framework and hypotheses development. This is followed by a detailed explanation of the research methodology, including questionnaire design, sampling procedures, and data analysis methods. Subsequently, the empirical results are reported and discussed, along with the theoretical and practical implications. Finally, the study's limitations and future research directions are spresented.

## 2 Theoretical framework and hypotheses development

### 2.1 SHC purchase intention

SHC purchase intention refers to consumers' willingness to purchase SHC products. As intention is widely recognized as a proximal predictor of behavior (Ajzen, 2002), extensive researchers studying SHC purchase behavior have examined SHC purchase intention (Kian et al., 2022; Madeline et al., 2023; Tryphena and Arul Aram, 2023). Recent studies have developed theoretical models to explain SHC purchase intention (Kian et al., 2022, 2023; Madeline et al., 2023; Nawaz Muhammad Zahid and Meng, 2023). While existing theoretical models examine SHC purchase intention, they primarily rely on generalized research frameworks rather than incorporating group-specific characteristics as key variables. To address this gap, this study employs the TPB framework to develop a comprehensive model for understanding the factors associated with SHC purchase intention among Chinese Gen Z consumers.

### 2.2 Literature review of theory

The TPB, originally formulated by Ajzen (1991), posits that behavioral intention serves as the central determinant of volitional action. According to this theoretical framework, purchase intention and, by extension, subsequent behavior can be reliably predicted through three principal constructs: attitude, perceived behavioral control (PBC), and subjective norms (Ajzen, 1991, 2002). The TPB has been widely applied to explain pro-environmental behaviors (Jun et al., 2022; Kian et al., 2022) and to predict purchase intentions related to various green products (e.g. organic food, energy efficient appliances etc.). In recent years, TPB has been widely applied in studies on sustainable clothing and SHC consumption (Kian et al., 2022; Madeline et al., 2023; Tewari et al., 2022; Tryphena and Arul Aram, 2023). However, while TPB has demonstrated robust predictive power in Western contexts, its applicability to non-Western cohorts, such as Chinese Gen Z consumers, remains underexplored. The TPB is generally conceptualized as a self-interest-based theoretical framework. Within this paradigm, all TPB variables function as rational choice predictors that conceptualize pro-environmental behavior as emerging from an individual's cost-benefit analysis (Abrahamse and Steg, 2009). Moore (1968) and Hansen (1983) posited that Chinese individuals perceive the world as an interconnected whole, placing particular emphasis on interpersonal relationships. Consequently, their analytical approach extends beyond isolated objects to incorporate broader contextual and environmental considerations. To address this gap, the study extends the traditional TPB model by integrating values and social influence to enhance its explanatory power in predicting SHC purchase intention within Chinese Gen Z consumers. By doing so, an exploratory test is provided by this research as to whether, in this specific sample, more variance is explained by the extended TPB framework than by the baseline model. Before proceeding to hypothesis development, it is important to clarify the conceptual boundaries of the three social influence constructs employed in this study: subjective norm, group norm, and social identity. Although all three originate from the broader social influence literature, they capture distinct mechanisms. Subjective norm (Ajzen, 1991) refers to perceived pressure from specific, personally important referents (e.g., family, close friends). Group norm (Yang, 2019) reflects the perceived alignment with the goals of a broader, less personally defined reference category (e.g., “SHC consumers” as a collective). Social identity (Bagozzi and Dholakia, 2002) concerns the internalized sense of belonging and value congruence with that group, motivating behavior to maintain a positive self-concept. Theoretically, these constructs address different questions: “What do important others think I should do?” (subjective norm), “What does the group I belong to value?” (group norm), and “To what extent do I see myself as part of this group?” (social identity). Despite their theoretical distinctions, the simultaneous inclusion of subjective norm, group norm, and social identity increases model complexity. We justify this choice on three grounds: (1) prior literature has called for disentangling different sources of social influence in sustainable consumption (Yang, 2019); (2) Chinese Gen Z's unique digital ecosystem may activate all three mechanisms differentially; and (3) Subjective norms, group norms, and social identity were integrated into a coherent framework by Yang (2019) without any apparent redundancy or potential multicollinearity issues.

### 2.3 Variables of TPB and hypotheses

According to Ajzen (1991), attitude is defined as the extent to which an individual holds a favorable or unfavorable evaluation regarding a specific behavior. His study suggests that positive attitudes toward a specific behavior are significantly associated with an increased likelihood of behavioral performance (Ajzen, 1991). When individuals perceive that a behavior will yield beneficial outcomes, they may develop a more favorable attitude toward it. Chinese Gen Z consumers, who have grown up during a period of rapid economic development and increasing environmental consciousness (Textor, 2022; Zhang et al., 2023), tend to exhibit more complex and nuanced attitudes toward sustainable consumption compared to earlier generations (Liang et al., 2022). Empirical studies consistently demonstrate that attitude is positively correlated with purchase intention in sustainable fashion and SHC contexts. For instance, Yadav and Pathak (2017) found that attitude is significantly and positively associated with green purchasing behavior among consumers in developing countries. Similarly, in studies more closely aligned with this research context, Kian et al. (2022) identified attitude as a robust determinant of SHC purchase intention, and Madeline et al. (2023) confirmed the predictive power of attitude on the SHC purchase intention. However, it should be noted that the strength of this relationship may vary depending on product type, consumer involvement, or cultural context (Rozenkowska, 2023). Building upon these established findings, the following hypothesis is proposed:

H1: Attitude is positively associated with Gen Z consumers' SHC purchase intention.

PBC is conceptually defined as an individual's self-assessment of the ease or difficulty associated with executing a specific behavior (Ajzen and Madden, 1986). Within the TPB framework, this construct has been empirically demonstrated to exhibit a positive correlation with behavioral intention, wherein heightened perceptions of control correspond to stronger intentions to engage in the target behavior (Ajzen, 1991). Chinese Gen Z consumers' noted digital proficiency and preference for convenient, reliable platforms (Pu et al., 2025) may significantly influence PBC over SHC transactions. In the context of sustainable fashion consumption, PBC has been widely recognized as a key psychological factor associated with consumer behavior. Prior research has demonstrated that consumers' perceived control over SHC purchases significantly enhances their purchase intention (Chaturvedi et al., 2020; Kian et al., 2022). Specifically, Chaturvedi et al. (2020) established PBC as a robust predictor of sustainable apparel adoption, and Kian et al. (2022) further corroborated its positive influence on SHC consumption behaviors. Based on this established theoretical foundation, the following hypothesis is proposed. Given that the predictive utility of PBC may be attenuated when the target behavior is perceived as easy or routine (Ajzen, 2002), H2 is proposed as an exploratory hypothesis.

H2: PBC is positively associated with Gen Z consumers' SHC purchase intention.

Subjective norm, defined as an individual's perception of social pressure to engage in or refrain from a particular behavior (Ajzen, 1991), has been consistently identified as a significant predictor of behavioral intention. Extensive empirical research has established the significant role of subjective norm in shaping purchase intention within sustainable fashion contexts (Kian et al., 2022). When individuals perceive that important referent groups approve of SHC consumption, this social influence serves as a powerful motivator for behavioral intention (Kian et al., 2022; Sobuj et al., 2021; Tewari et al., 2022). Kian et al. (2022) identified a positive correlation between subjective norm and SHC purchase intention. Sobuj et al. (2021) specifically demonstrated subjective norm influence among young Bangladeshi consumers' purchase intention to eco-friendly apparel. Tewari et al. (2022) found that subjective norm positively related to consumers' purchase intention to green apparel. Given the rapid expansion of social e-commerce platforms in China has amplified the impact of peer influence (Sun et al., 2022). Gen Z, in particular, has been found to be more responsive to social media trends and influencer endorsements (Sun et al., 2022). These unique social dynamics suggest that the consumption behaviors of Chinese Gen Z consumers may be especially vulnerable to normative pressures. The greater the perceived support from peers, influential individuals, and key referent groups regarding SHC purchases, the higher the likelihood that Gen Z consumers will engage in such behavior. Simultaneously acknowledging that the influence of subjective norm may be moderated by individual differences such as need for uniqueness or autonomy (Batra et al., 2001). Building upon this robust empirical foundation, we posit the following hypothesis.

H3: Subjective norm is positively associated with Gen Z consumers' SHC purchase intention.

### 2.4 Values and hypotheses

Biospheric values, as defined by Stern (2000), reflect consumers' prioritization of sustainability and environmental protection when making purchasing decisions. Biospheric values emphasize the preservation and respect for entire ecosystems, serving as a fundamental motivator for environmental behaviors (Ünal et al., 2019). Extensive research has documented positive correlations between biospheric values and various environmental behaviors. In the domain of pro-environmental behaviors, Sanchez-Garcia et al. (2021) confirmed their positive association with behavioral intention. This relationship has been further validated in sustainable consumption research, with Yadav et al. (2019) demonstrating its impact on green hotel selection and identifying attitude as a mediating variable. More recently, Bhardwaj et al. (2023) reinforced these findings, identifying biospheric values as primary correlates of both green purchase attitude and intention.

The biospheric values examined in this study reflect the extent to which Chinese Gen Z consumers prioritize sustainable development and environmental protection when purchasing SHC. Chinese traditional Confucian values emphasize harmonious unity of human and nature (Wang et al., 2022). Furthermore, China's distinctive environmental policy landscape, characterized by ambitious carbon neutrality goals (Chen et al., 2024), has fostered unique sustainability awareness among younger consumers. Compared to previous generations, Chinese Gen Z demonstrates greater concern for environmental issues (Kim et al., 2020). Consequently, it is plausible that higher biospheric values, such as a commitment to sustainability and ecological preservation, are associated with more favorable attitudes and stronger purchase intentions. Based on this substantial empirical evidence, the following hypothesis is proposed:

H4: Biospheric values are positively associated with Gen Z consumers' SHC purchase intention.

H5: Biospheric values are positively associated with Gen Z consumers' attitude.

Altruistic values, conceptualized as consumers' concern for collective welfare manifested through purchasing decisions, have been extensively studied as correlates of pro-environmental behavior (Stern, 2000). Extensive empirical research has documented positive correlations between altruistic values and pro-environmental behaviors. It has been demonstrated by multiple studies that individuals who emphasize altruistic values are more likely to exhibit stronger environmental beliefs and engage in pro-environmental behaviors (Albayrak et al., 2011). Sanchez-Garcia et al. (2021) demonstrated the positive association of altruistic values on behavioral intentions, while Birch et al. (2018) confirmed their impact on attitude toward green products. Subsequent studies have consistently validated these findings across various sustainable consumption contexts. Wang et al. (2020) identified significant positive associations with green hotel selection. Yadav (2016) established their determining role in organic food purchases among young consumers. Reimers et al. (2017) found them to be the most important factor associated with attitude toward sustainable clothing. Bhardwaj et al. (2023) reinforced their primary role in green purchase decisions. Haj-Salem et al. (2022) confirmed their positive association on green product attitude. Kucher et al. (2019) demonstrated correlation with willingness to pay for pro-social products.

The altruistic values examined in this study reflect the concern of Chinese Gen Z for the wellbeing of others and demonstrate a commitment to social welfare through the purchase of SHC. Chinese Gen Z exhibits heightened sensitivity to social issues (Nanda, 2020) and may possess a stronger sense of moral obligation to contribute to societal betterment. Consequently, it is plausible that higher altruistic values such as promoting social welfare and assisting others, are associated with more favorable attitudes and stronger purchase intentions. Based on this comprehensive body of evidence, the following hypothesis is proposed:

H6: Altruistic values are positively associated with Gen Z consumers' SHC purchase intention.

H7: Altruistic values are positively associated with Gen Z consumers' attitude.

### 2.5 Social influence and hypotheses

Social influence refers to the extent to which individuals consider others' opinions when making behavioral decisions (Hu et al., 2019). As social beings, people's perceptions and actions are inevitably associated with interpersonal interactions within complex social networks (Friedkin, 1998; Zhang et al., 2014). Particularly in the context of Gen Z consumers' intention to purchase sustainable clothing products (Ngo et al., 2024), the views and behaviors of significant referent groups exert considerable influence on individual decision-making processes. Three primary dimensions of social influence have been identified in the literature: subjective norm, group norm, and social identity (Yang, 2019). In this section, we first restate the hypothesis for subjective norm (already developed in the TPB section) and then develop hypotheses for group norm and social identity, providing explicit theoretical justifications for their inclusion.

Subjective norm, as defined by Ajzen (1991), is conceptualized as the perceived social pressure exerted by specific referent individuals. Given the collectivist orientation of Chinese society and the amplified peer influence through social media (Sun et al., 2022), we retain this construct as a baseline social influence variable.

Group norm, defined as the perceived goal alignment with a broader reference category of “SHC consumers” (Yang, 2019), is conceptually distinct from subjective norm because the referent is not personally known individuals but an abstract social category. The group norm dimension is particularly relevant for Chinese Gen Z consumers, who, as digital natives, actively participate in and are simultaneously represented by social media communities (Tsai and Bagozzi, 2014). For Chinese Gen Z consumers, who actively participate in platform-based communities (e.g., Xiaohongshu, Douyin), the normative alignment with such digital communities may operate through value congruence rather than explicit pressure (Tan, 2024). Within these digital communities, the value alignment process (Kim and Park, 2011) is often manifested through the viral dissemination of challenges such as old clothes new wear, by which distinct normative pressures are generated. This construct captures a more diffuse, community-level influence that subjective norm does not address. A positive correlation between group norms and purchase intention has been reported in previous research on ethical consumption, most notably by Nenci et al. (2008) in the context of ethnic food products. However, the effect of group norm may depend on the perceived cohesiveness and relevance of the reference group. When individuals belong to multiple, fluid online communities, normative pressure from any single group may be diluted (Chih et al., 2017). Building upon this robust empirical foundation, H8 is proposed as an exploratory hypothesis.

H8: Group norm is positively associated with Gen Z consumers' purchase intention.

Social identity, defined as the internalized sense of belonging and value congruence with a group (Bagozzi and Dholakia, 2002), captures the motivational force derived from self-definition. This psychological construct motivates individuals to align their actions with group expectations, thereby preserving favorable social connections (Yang, 2019). Among Chinese Gen Z, the intersection of collectivist traditions and individualistic expressions creates complex identity negotiation (Xu and Gong, 2024). Social identity may be associated with SHC purchase intention through self-consistency and group-based self-esteem, mechanisms distinct from normative pressure. Group norm focuses on perceived goal alignment, whereas social identity focuses on self definition and emotional belonging. These contextual specificities justify the need to examine the influence of social identity on purchase intention in the Chinese SHC market. Prior research has reported positive correlations between social identity and sustainable consumption intentions (Zheng et al., 2023). Based on this theoretical and empirical foundation, we propose:

H9: Social identity is positively associated with Gen Z consumers' purchase intention.

Nevertheless, the strength of this relationship likely varies with the individual's degree of identification with the group (Tajfel and Turner, 1979). As with all hypotheses, H9 is tested as an associational pattern, not a causal claim.

**Figure 1:**
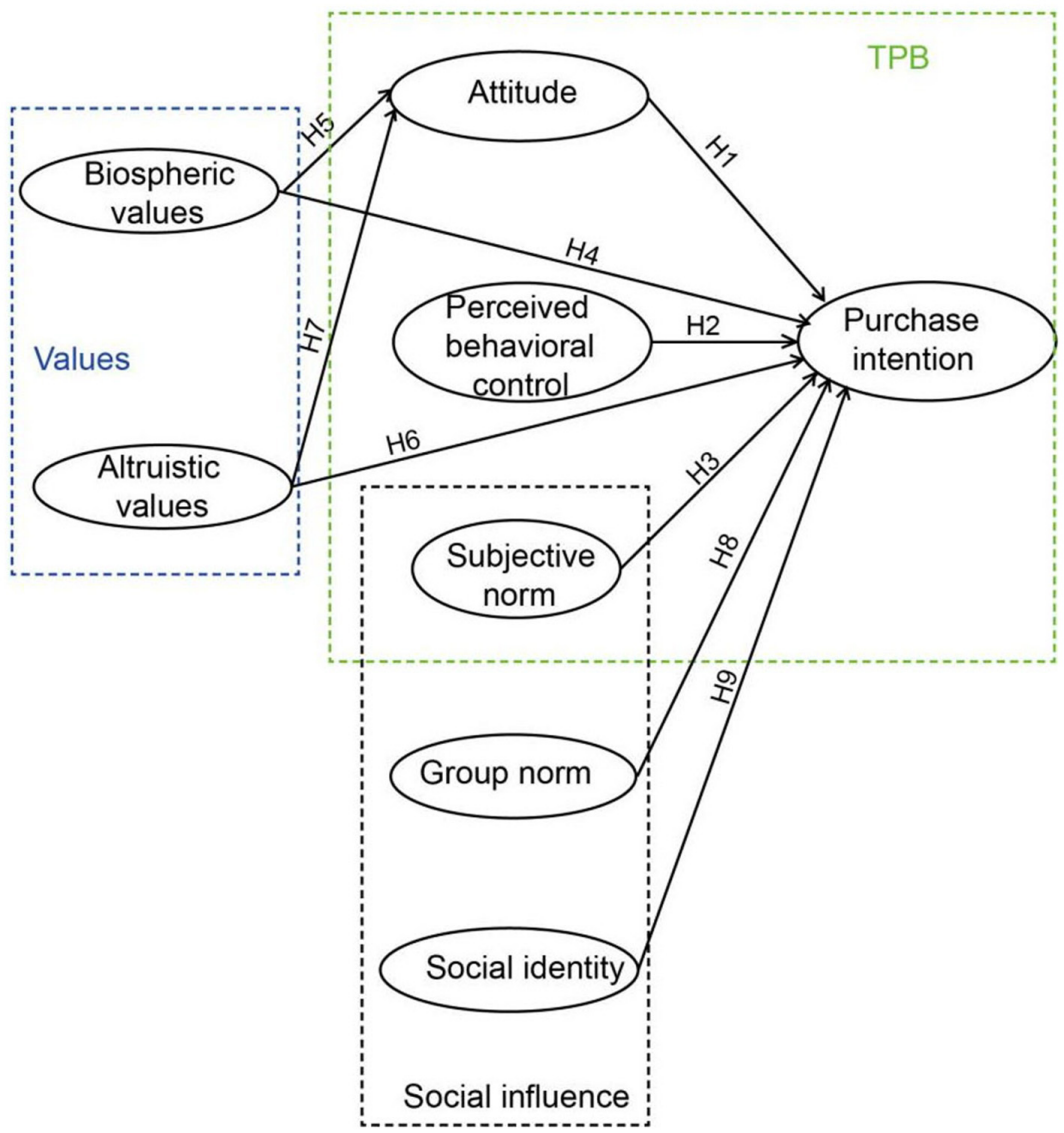
Comprehensive model.

Building upon these theoretical foundations, we propose an integrated conceptual framework (Figure 1) to examine the correlates of SHC purchase intention among Chinese Gen Z consumers.

## 3 Methods

### 3.1 Question design

The research instrument comprised three distinct sections designed to collect targeted data. Section one served as a screening mechanism, employing the qualifying question: “Were you born between 1997 and 2012 and are currently aged 18 or above?” Only respondents meeting these demographic criteria proceeded to subsequent sections. Section two gathered fundamental demographic characteristics and participants' prior experience with SHC purchases. In the third section, validated measurement scales were incorporated, primarily using a five-point Likert format (1 = strongly disagree to 5 = strongly agree) unless otherwise noted. These scales included measures of altruistic values (Sanchez-Garcia et al., 2021; Sun et al., 2024), biospheric values (Sanchez-Garcia et al., 2021), attitude (Ajzen, 2002), subjective norm (Tu and Hu, 2018), PBC (Petkowicz et al., 2024), group norm (Yang, 2019), social identity (Yang, 2019) and SHC purchase intention scales (Kian et al., 2022). All measurement items were adapted from established instruments in prior studies, with modifications made to ensure contextual relevance while maintaining construct validity. Social identity was operationalized as the perceived degree of overlap between the self and the group of SHC consumers. This construct was measured using a five-point pictorial scale, with items SI1 and SI2 each presenting respondents with a series of five paired-circle diagrams. These diagrams ranged from two entirely separate circles (scored as 1, indicating “no overlap”) to two nearly concentric circles (scored as 5, indicating “almost complete overlap”). Participants were instructed to select the image pair that best visualized their own perceived level of identity integration with the consumer group. To ensure data quality, two attention-check questions (“Please select ‘1' for this item” and “Please select ‘5' for this item”) were embedded in the third section, along with two consistency validation items (“Peace in the world is unimportant to me” and “I do not feel obliged to make any effort toward building a beautiful world”) designed to detect inconsistencies in responses to reversed items. It should be noted that group norm was measured using a two-item scale adapted from Yang (2019). Although multi-item scales are common, a two-item measure is justified given the unidimensional nature of the construct, strong inter-item correlation (*r* = 0.71, *p* < 0.01), and satisfactory reliability as indicated by the Spearman-Brown coefficient (ρ =0.830) and factor loading (>0.7 in CFA). Prior research has successfully employed two-item scales for similar normative constructs (Meiksin et al., 2024). Moreover, limiting the number of items helped reduce survey length and participant fatigue, thereby enhancing data quality in this field setting. However, the use of a two-item scale remains a measurement limitation, which we acknowledge in the Limitations section. A rigorous translation protocol was implemented, involving bidirectional translation (English–Chinese–English) by two bilingual researchers to guarantee linguistic equivalence. The researchers consulted four doctors to get their feedback on the questionnaire. Thirty volunteers were recruited to answer the questionnaire to verify its reliability, and some of the unclear wording was changed after they made comments. Table 1 shows the scale items.

**Table 1:** Results of reliability and convergent validity.

Constructs and items	Factor loadings	AVE	CR	α
Biospheric values		0.603	0.821	0.815
BV1: I respect the earth	0.734			
BV2: I am an integral part of nature	0.742			
BV3: I should protect nature and environment	0.778			
BV4: I should make effort to build a beautiful world	0.753			
Altruistic values		0.621	0.838	0.841
AV1: Men are created equal	0.731			
AV2: Society should be justice	0.784			
AV3: The world should be peace	0.749			
AV4: I should be an altruist	0.773			
Attitude		0.628	0.855	0.862
ATT1: It is good to purchase SHC	0.772			
ATT2: It is pleasant to purchase SHC	0.752			
ATT3: It is valuable to purchase SHC	0.813			
ATT4: It is beneficial to purchase SHC	0.778			
Subjective norm		0.613	0.841	0.845
SN1: The people who can influence my decision-making support me to purchase SHC	0.762			
SN2: Those who are important to me support me to purchase SHC	0.823			
SN3: Those I am concerned about support me to purchase SHC	0.783			
Perceived behavioral control		0.629	0.862	0.857
PBC1: I am confident that I can purchase SHC whenever I want	0.787			
PBC2: I see myself prepared to purchase SHC in the future	0.802			
PBC3: I have the resources, time and availability to purchase SHC	0.813			
PBC4: I probably have a lot of chances to purchase SHC	0.759			
PBC5: The decision to purchase SHC is entirely up to me	0.794			
Group norm (Buying SHC in the next purchase is regarded as a goal. Please estimate the power to which each achieves the goal)		0.591	0.740	0.744
GN1: Power of self's goal	0.810			
GN2: Average of the power of SHC consumers' goal	0.732			
Social identity		0.613	0.818	0.827
SI1:When you are actually a SHC consumer, how would you express the degree of overlap between your personal identity and identity of SHC?	0.765			
SI2: Please indicate to what extent your self-image overlaps with your identity as a SHC consumer	0.783			
SI3: How attached are you to SHC?	0.794			
SI4: How strong is your sense of belonging to SHC consumers?	0.758			
SI5: I am a valuable member of SHC consumers	0.803			
SI6: I am an important member of SHC consumers	0.791			
Purchase intention		0.663	0.797	0.798
PI1: I'm very likely to purchase SHC in the future	0.811			
PI2: Certainly, I will purchase SHC	0.817			

Group norm and purchase intention were measured with two items; Spearman-Brown coefficients were 0.830 and 0.797, respectively, and inter-item correlations were 0.71 and 0.73 (*p* < 0.01).

### 3.2 Sample

Data collection was conducted via an online survey platform (www.wenjuan.com) between January and March 2025. Prior to participation, all respondents were presented with a research information sheet on the first page of the online survey. The information sheet outlined the purpose of the study, the voluntary nature of participation, data confidentiality measures, and the right to withdraw at any time without consequence. By voluntarily proceeding to complete the survey, participants implied their willingness to participate. The survey was disseminated through multiple digital channels, including email, WeChat, and Sina Weibo, targeting Chinese Gen Z adults as the specific population of interest.

To enhance sample diversity across different regional contexts, a non-probability stratified quota sampling approach was employed. The population was stratified according to city tier (first-tier, second-tier, and third-tier or below), a well-established classification that captures meaningful variations in economic development, consumer infrastructure, and lifestyle patterns across China. A target of 300–400 valid responses was set for each stratum to ensure sufficient statistical power for subgroup analyses. Because a non-probability stratified quota sampling method was employed in this study, the final sample was not weighted to population parameters. Consequently, the findings are not statistically generalizable to the entire Chinese Gen Z population, rather, they represent associational patterns observed within a large but non-representative sample. Although the quota design ensures that substantial variation across city tiers is captured by the sample—which may support the theoretical generalizability of the observed relationships (i.e., the patterns may hold across different urban contexts)—this remains an empirical question to be addressed in future research.

From an initial pool of 1,075 responses, data screening was conducted as follows. First, 71 respondents who failed at least one attention-check question were removed. Second, 37 respondents were excluded due to any inconsistent logic in their responses. Inconsistent responding was defined as an absolute difference of two points or greater on the five-point Likert scale between the original item and a subsequently reversed item. After these exclusions, 967 valid responses remained. Valid responses were distributed across city tiers as follows: 331 (first-tier), 325 (second-tier), and 311 (third-tier or below). The sample demographics were characterized as follows: 41.6% of respondents were male, and 58.4% of respondents were female. 10.9% of participants indicated that their education level was below undergraduate, 36.3% of respondents revealed that they were undergraduate students, 38.8% of respondents indicated that they were graduates, and others were postgraduates. Approximately 38.5% of respondents reported having SHC purchase experience.

### 3.3 Data analysis

This study employed a cross-sectional, self-report survey design with a non-probability stratified quota sampling. Cross-sectional designs capture relationships at a single point in time and cannot establish causal precedence or temporal ordering.

Structural equation modeling (SEM) was employed as the analytical framework to examine the hypothesized associations within the proposed theoretical model. SEM is a robust multivariate technique that offers several advantages over conventional multiple regression for examining complex interrelationships among variables (Wang et al., 2022). First, it accounts for measurement error by modeling latent constructs through multiple observed indicators, thereby enhancing the validity of the measurement (Wang et al., 2022). Second, its simultaneous equation estimation approach helps mitigate endogeneity concerns and potential bias from reciprocal causation (Cao and Yang, 2017). Therefore, we use SEM to examine our research hypothesis as associational patterns. The analysis comprises two main components: the measurement model and the structural model. The measurement model evaluates the relationships between observed indicators and their underlying latent constructs, while the structural model defines associations between unobserved latent structures.

In our study, we used SPSS (version 26) and AMOS (version 24) to analyze collected data. The following analytical steps were performed in order: (1) descriptive statistics and reliability analysis (Cronbach's alpha) using SPSS; (2) confirmatory factor analysis (CFA) using AMOS with maximum likelihood (ML) estimation to assess measurement model fit, convergent validity (AVE), and discriminant validity (Fornell–Larcker and HTMT); and (3)SEM using AMOS with ML estimation to test the hypothesized paths. In addition, we conduct modeling comparisons to compare the in-sample explanatory performance of the proposed theoretical framework against the baseline TPB model.

To examine the robustness of the structure model. We explore whether the hypothesized relationships varied across key demographic and city-tier subgroups, multi-group SEM was conducted. Two grouping variables were examined: gender (male vs. female), city tier (first-tier, second-tier, third-tier or below). For each grouping variable, a series of nested models was compared. First, an unconstrained model was estimated, in which all structural paths were allowed to differ across groups. Second, a constrained model was estimated, in which each path of interest (e.g., social identity → purchase intention) was forced to be equal across groups. A significant chi-square difference test (Δχ^2^) between the constrained and unconstrained models indicated that the path coefficient differed significantly between subgroups (Byrne, 2016). For city-tier comparisons, given the involvement of three groups, pairwise comparisons were conducted following a significant overall omnibus test.

## 4 Results

### 4.1 Measurement model

First, SPSS software was used to evaluate the scale's reliability and validity. Cronbach's alpha coefficients ranged from 0.744 to 0.862 (>0.7), and the Kaiser–Meyer–Olkin (KMO) value was 0.863 (>0.5). These results indicated that acceptable internal consistency reliability was demonstrated by all constructs and that the overall adequacy of the sample was acceptable (Hair et al., 2012).

A key premise of SEM analysis is that the data approximate multivariate normality (Kline, 2016). Therefore, multivariate normality was assessed by first examining univariate skewness and kurtosis for all scale items, followed by the Mardia's multivariate kurtosis coefficient and its associated critical ratio (C.R.). Univariate skewness values for all items ranged from −1.22 to 1.06 (all < |2|), and univariate kurtosis values ranged from −0.92 to 1.74 (all < |4|), conforming to normal distribution assumptions (Field, 2013). Mardia's multivariate kurtosis was estimated at 2.94, with a critical ratio (C.R.) of 1.52, which is below the recommended threshold of 1.96 (*p* > 0.05), indicating that the multivariate normality assumption was not violated.

CFA was conducted using AMOS with ML estimation. All factor loadings in the range of 0.731–0.823 (>0.7) and composite reliability (CR) values in the range of 0.740–0.862 (>0.7), which were both above the recommended threshold, indicating the internal consistency and reliability were all good (in Table 1). Moreover, the average variance extracted (AVE) values ranged from 0.591 to 0.663 (>0.5), signifying that the model had good convergent effectiveness (Bagozzi and Yi, 1988). Discriminant validity was first assessed using the Fornell–Larcker criterion. The square root of each AVE (ranging from 0.769 to 0.814) was greater than the corresponding inter-construct correlations (see Table 2), meeting the required standard (Hair et al., 2017). To provide stronger evidence, the heterotrait–monotrait (HTMT) ratio of correlations was additionally calculated. All HTMT values were below the conservative threshold of 0.85 (see Table 2), confirming discriminant validity (Henseler et al., 2015). The model's χ^2^ = 538, df = 316, *p* < 0.001, and the χ^2^/df value was 1.703 (< 3). In addition, the values of the fit indicators, namely GFI, AGFI, CFI, and TLI, were found to be 0.937, 0.945, 0.963, and 0.968 (all > 0.9), respectively. And the RMSEA value is 0.032 (< 0.08), all indicating an excellent model fit (Rönkkö and Cho, 2022).

**Table 2:** Discriminant validities.

Constructs	BV	AV	ATT	SN	PBC	GN	SI	PI
Fornell and Larcker criterion								
BV	0.777							
AV	0.238	0.788						
ATT	0.232	0.225	0.792					
SN	0.087	0.096	0.154	0.783				
PBC	0.103	0.083	0.039	0.028	0.793			
GN	0.132	0.103	0.097	0.128	0.028	0.769		
SI	0.067	0.045	0.119	0.128	0.094	0.202	0.783	
PI	0.251	0.213	0.231	0.149	0.206	0.121	0.427	0.814
Heterotrait-Monotrait criterion								
BV								
AV	0.288							
ATT	0.281	0.248						
SN	0.118	0.120	0.189					
PBC	0.129	0.104	0.049	0.035				
GN	0.164	0.130	0.131	0.162	0.035			
SI	0.084	0.054	0.147	0.162	0.118	0.252		
PI	0.314	0.286	0.292	0.180	0.249	0.155	0.541	

BV, biospheric values; AV, altruistic values; ATT, attitude; SN, subjective norm; PBC, perceived behavioral control; GN, group norm; SI, social identity; PI, purchase intention.

Given that all data were collected via self-report questionnaires at a single time point, common method bias (CMB) was a potential concern. To assess the severity of CMB, two complementary approaches were employed. First, Harman's single-factor test was conducted by entering all measurement items into an unrotated exploratory factor analysis. The results showed that the first factor accounted for 25.713% of the total variance, well below the recommended threshold of 50%, suggesting that CMB was not a pervasive issue. Second, an unmeasured latent method factor (ULMC) approach was employed within the CFA framework. All indicators were allowed to load on both their respective trait constructs and a common method factor. The inclusion of the method factor resulted in negligible improvements in model fit (ΔCFI = 0.008, ΔRMSEA = 0.004), as shown in Table 3. Furthermore, as reported in Table 4, none of the method factor loadings were statistically significant (all *p* > 0.05), with individual loadings ranging from 0.009 to 0.095 (average = 0.058). These findings indicate that common method bias did not substantially influence the results (Podsakoff et al., 2012). Additionally, the variance inflation factor (VIF) was used to measure the level of multicollinearity between the latent constructs (Ahmed et al., 2024). As shown in Table 5, VIF values for the independent variables ranged from 1.165 to 1.308, all well below the conservative threshold of 2, confirming the absence of multicollinearity. Taken together, these analyses support the validity and reliability of the structural estimates for this sample.

**Table 3:** Comparison of model fit with and without unmeasured latent method factor (ULMC).

Model	*χ^2^*	df	χ^2^/df	CFI	RMSEA	ΔCFI	ΔRMSEA
Measurement model (without method factor)	538.00	316	1.703	0.963	0.032	–	–
Measurement model (with method factor)	521.34	286	1.823	0.971	0.036	+0.008	+0.004

**Table 4:** Standardized factor loadings of the common method factor (ULMC).

Construct	Item	Loading on trait construct (range)	Loading on method factor (range)	*p*-Value (method loading)
BV	BV1–BV4	0.734 – 0.778	0.021–0.087	0.213–0.468
AV	AV1–AV4	0.731–0.784	0.015–0.092	0.187–0.512
ATT	ATT1–ATT4	0.752–0.813	0.034–0.078	0.156–0.394
SN	SN1–SN3	0.762–0.823	0.018–0.065	0.224–0.487
PBC	PBC1–PBC5	0.759–0.813	0.009–0.071	0.301–0.623
GN	GN1–GN2	0.732–0.810	0.041–0.083	0.178–0.352
SI	SI1–SI6	0.758–0.803	0.027–0.095	0.132–0.415
PI	PI1–PI2	0.811–0.817	0.032–0.069	0.245–0.389

BV, biospheric values; AV, altruistic values; ATT, attitude; SN, subjective norm; PBCl, perceived behavioral control; GN, group norm; SI, social identity; PI, purchase intention.

**Table 5:** Path coefficients and direct associations for hypothesis.

Relations	Path coefficient	**S.E**.	*p*-values	Result	VIF
ATT → PI	0.168	0.037	0.000	H1 supported	1.165
PBC → PI	0.059	0.047	0.252	H2 not supported	1.179
SN → PI	0.187	0.041	0.000	H3 supported	1.274
BV → PI	0.143	0.042	0.001	H4 supported	1.283
BV → ATT	0.201	0.045	0.000	H5 supported	1.283
AV → PI	0.154	0.041	0.002	H6 supported	1.308
AV → ATT	0.197	0.047	0.000	H7 supported	1.308
GN → PI	0.147	0.145	0.310	H8 not supported	1.229
SI → PI	0.394	0.056	0.000	H9 supported	1.196

Good-of-fit statistics for the Integrated model.

χ2 =2854.473 (df = 986, p < 0.001), CFI = 0.908, TLI = 0.943, SRMR = 0.057, RMSEA = 0.043.

BV, biospheric values; AV, altruistic values; ATT, attitude; SN, subjective norm; PBC, perceived behavioral control; GN, group norm; SI, social identity; PI, purchase intention.

While the measurement model was found to meet conventional thresholds, several caveats warrant consideration. First, despite the theoretical, empirical, and practical arguments supporting two-item scales, it should be noted that the two-item measures of group norm and purchase intention may not fully capture the breadth of these constructs and are more susceptible to measurement error (Kline, 2016). Second, different response formats were employed (a five-point Likert scale for most constructs and a pictorial scale for social identity), a practice that could introduce method effects, though the ULMC test indicated no systematic bias.

### 4.2 Structural model

The structural model was estimated using AMOS with ML estimation. The results showed that the proposed integrated model exhibited higher in-sample explanatory performance and higher goodness-of-fit indices than the baseline TPB model. Specifically, the integrated model demonstrated improved goodness-of-fit indices (χ^2^ = 2854.473; df = 986; *p* < 0.001; χ^2^/df = 2.895; CFI = 0.908; TLI = 0.943; SRMR = 0.057; RMSEA = 0.043) compared to the TPB model (χ^2^ = 708.946, df = 158, *p* < 0.001; χ^2^/df = 4.487; CFI = 0.856; TLI = 0.925; SRMR = 0.071; RMSEA = 0.056). Furthermore, the integrated model explained 53.7% of the variance in SHC purchase intention, whereas the TPB model explained 34.2%. An intermediate model combining TPB and values explained 43.6% of the variance. Thus, within the current sample, the integrated model explained substantially more variance than the TPB baseline.

Table 5 presents the hypothesis testing results. Consistent with the associational nature of this study, we report standardized coefficients (β) as measures of the strength of association, not as causal effects. Attitude (β = 0.168, *p* < 0.001) and subjective norm (β = 0.187, *p* < 0.001) are positively associated with purchase intention, thus H1 and H3 were supported. But the association between PBC and purchase intention was not statistically significant (β = 0.059, *p* > 0.1), thus H2 was not supported. Both biospheric values (β = 0.143, *p* < 0.01) and altruistic values (β = 0.154, *p* < 0.01) are positively associated with Gen Z consumers' SHC purchase intention. Therefore, H4 and H6 were supported. The hypothesized associations of both biospheric values and altruistic values with attitude were examined. Results reveal that biospheric values (β = 0.201, *p* < 0.001) and altruistic values (β = 0.197, *p* < 0.001) are positively associated with attitude. Therefore, H5 and H7 were supported. Social identity is positively associated with purchase intention (β = 0.394, *p* < 0.001), Therefore, H9 was supported. Group norm was not significantly associated with purchase intention (β = 0.147, *p* > 0.1), thus, H8 was not supported.

Multi-group SEM was performed to examine whether the hypothesized structural paths differed by gender and by city tier. The sample sizes for each subgroup were as follows: male (*n* = 402), female (*n* = 565); first-tier cities (*n* = 331), second-tier cities (*n* = 325), and third-tier or below cities (*n* = 311). The key path coefficients across subgroups are summarized in Table 6.

**Table 6:** Multi-group comparisons of structural paths by gender and city tier.

Path	Comparison	Unconstrained β (group A)	Unconstrained β (group B)	Δχ^2^ (df = 1)	*p*-Value
Gender (male vs. female)		Male (*n* = 402)	Female (*n* = 565)		
ATT → PI	Male vs. female	0.160	0.173	0.18	0.67
SN → PI	Male vs. female	0.179	0.192	0.21	0.65
BV → PI	Male vs. female	0.137	0.149	0.28	0.60
BV → ATT	Male vs. female	0.195	0.207	0.31	0.58
AV → PI	Male vs. female	0.148	0.158	0.22	0.64
AV → ATT	Male vs. female	0.191	0.203	0.26	0.61
SI → PI	Male vs. female	0.381	0.402	0.54	0.46
City tier (first-tier vs. second-tier)		First (*n* = 331)	Second (*n* = 325)		
ATT → PI	First-tier vs. second-tier	0.155	0.168	0.15	0.70
SN → PI	First-tier vs. second-tier	0.172	0.184	0.12	0.73
BV → PI	First-tier vs. second-tier	0.141	0.153	0.19	0.66
BV → ATT	First-tier vs. second-tier	0.199	0.211	0.22	0.64
AV → PI	First-tier vs. second-tier	0.152	0.162	0.14	0.71
AV → ATT	First-tier vs. second-tier	0.194	0.205	0.17	0.68
SI → PI	First-tier vs. second-tier	0.385	0.391	0.06	0.81
City tier (first-tier vs. third-tier or below)		First (*n* = 331)	Third (*n* = 311)		
ATT → PI	First-tier vs. third-tier	0.155	0.179	0.42	0.52
SN → PI	First-tier vs. third-tier	0.172	0.198	0.54	0.46
BV → PI	First-tier vs. third-tier	0.141	0.164	0.38	0.54
BV → ATT	First-tier vs. third-tier	0.199	0.215	0.25	0.62
AV → PI	First-tier vs. third-tier	0.152	0.171	0.29	0.59
AV → ATT	First-tier vs. third-tier	0.194	0.212	0.30	0.58
SI → PI	First-tier vs. third-tier	0.385	0.408	0.33	0.57
City tier (second-tier vs. third-tier or below)		Second (*n* = 325)	Third (*n* = 311)		
ATT → PI	Second-tier vs. third-tier	0.168	0.179	0.10	0.75
SN → PI	Second-tier vs. third-tier	0.184	0.198	0.18	0.67
BV → PI	Second-tier vs. third-tier	0.153	0.164	0.21	0.65
BV → ATT	Second-tier vs. third-tier	0.211	0.215	0.08	0.78
AV → PI	Second-tier vs. third-tier	0.162	0.171	0.16	0.69
AV → ATT	Second-tier vs. third-tier	0.205	0.212	0.12	0.73
SI → PI	Second-tier vs. third-tier	0.391	0.408	0.15	0.70

ATT, attitude; SN, subjective norm, BV, biospheric values; AV, altruistic values; SI, social identity; PI, purchase intention.

All Δχ^2^ values are from chi-square difference tests between constrained (path equal across groups) and unconstrained models. No adjustments for multiple comparisons were applied. For city-tier comparisons, pairwise comparisons were conducted following an overall omnibus test. All p-values > 0.05 indicate no significant subgroup differences.

No significant differences were found between male and female respondents for any of the structural paths, including the associations between biospheric values and purchase intention (BV → PI: β_male_= 0.137, β_female_ = 0.149; Δχ^2^ = 0.28, *p* = 0.60), altruistic values and purchase intention (AV → PI: β_male_ = 0.148, β_female_ = 0.158; Δχ^2^ = 0.22, *p* = 0.64), biospheric values and attitude (BV → ATT: β_male_ = 0.195, β_female_ = 0.207; Δχ^2^ = 0.31, *p* = 0.58), and altruistic values and attitude (AV → ATT: β_male_ = 0.191, β_female_ = 0.203; Δχ^2^ = 0.26, *p* = 0.61). All other paths also showed no significant gender differences (all Δχ^2^ *p* > 0.05). Thus, the overall pattern of associations was largely invariant across genders in this sample.

No significant differences were found across first-tier, second-tier, and third-tier or below cities for any of the structural paths, including those involving biospheric and altruistic values. For example, the associations between biospheric values and purchase intention were β = 0.141 in first-tier cities, β = 0.153 in second-tier cities, and β = 0.164 in third-tier or below cities. None of the pairwise differences were statistically significant (first vs. second: Δχ^2^ = 0.19, *p* = 0.66; first vs. third: Δχ^2^ = 0.38, *p* = 0.54; second vs. third: Δχ^2^ = 0.21, *p* = 0.65). Similarly, the associations between altruistic values and attitude were β = 0.194 in first-tier cities, 0.205 in second-tier cities, and 0.212 in third-tier or below cities, with all pairwise comparisons being nonsignificant (all *p* > 0.05). All other hypothesized paths also showed no significant differences across city tiers. These findings suggest that the structural relationships are generally consistent across urban contexts of varying economic development levels, and that the role of values (biospheric and altruistic) in shaping attitude and purchase intention does not vary substantially by city tier.

Collectively, the multi-group analyses indicated that the structural relationships were largely invariant across gender and across city tiers. No statistically significant differences were detected for any of the hypothesized paths when comparing male with female respondents, nor when comparing first-tier, second-tier, and third-tier or below cities. The consistency of the core findings across these demographic and geographic subgroups suggests that the observed associations are robust and not substantially influenced by gender or regional economic context, thereby validating the robustness of the research results.

## 5 Discussion and implications

### 5.1 Discussion

This study proposed and empirically tested an extended model to examine factors correlated with Chinese Gen Z consumers' intention to purchase SHC.

Contrary to expectations, subjective norm (β = 0.187) showed a slightly stronger association with purchase intention than attitude (β = 0.168). One plausible interpretation is that this pattern reflects the collectivist cultural framework in which Chinese consumers are socialized, where the perceived expectations of significant others (family, close friends, and key opinion leaders) may carry substantial weight in socially visible consumption decisions. However, this interpretation must be regarded as tentative, as alternative explanations cannot be ruled out. The small difference of the coefficients suggest that the apparent dominance of subjective norm may not be statistically meaningful. Moreover, common method bias or measurement error may have differentially affected the two constructs. Thus, although the finding is suggestive of cultural influence, it should not be over-interpreted as strong evidence for the superiority of normative pressure over personal attitude, given that direct cross-cultural comparisons were not conducted in the present study. A noteworthy finding is the statistically non-significant relationship between PBC and purchase intention (*p* > 0.05), which diverges from several prior studies (Chaturvedi et al., 2020; Kian et al., 2022; Tewari et al., 2022). The mean PBC score was 4.07 (SD = 0.52; skewness = −0.54, kurtosis = −0.21), indicating a moderately left-skewed distribution and a potential ceiling effect. One possible explanation is that Chinese Gen Z consumers are highly proficient with e-commerce and online resale platforms, perceiving only minimal barriers (such as access, procedural knowledge, or logistical difficulty) to the acquisition of SHC. Consequently, variation in PBC may not differentiate purchase intention within this group. This interpretation aligns with the principle that the predictive utility of TPB constructs is context-dependent (Ajzen, 2002). Nevertheless, several alternative explanations warrant consideration. First, the non-significant effect could arise from insufficient variance in PBC scores (SD = 0.52) or from the possibility that PBC is more relevant for behaviors involving substantial obstacles, which SHC purchasing may not present for this digitally fluent cohort. Second, the null finding could reflect a genuine theoretical boundary: for routine, low-effort behaviors, perceived control may simply not be a discriminating factor. Given these possibilities, the nonsignificant result should be interpreted with caution, and replication in samples with greater PBC variability is needed.

Contrary to hypotheses, the nonsignificant effect of group norms diverges from expectations derived from social influence theory. The mean group norm score was 3.42 (SD = 0.91; skewness = −0.12, kurtosis = −0.35), indicating a relatively normal distribution. One plausible explanation is that Gen Z consumers inhabit pluralistic digital social ecosystems, navigating multiple fluid online communities with diverse values. As a result, normative pressure from any single group may be diluted, and thus may have a weaker association with personally expressive consumption choices such as SHC. However, this post hoc interpretation should be treated with caution. Several alternative explanations warrant consideration. First, the group norm construct was measured using only two items (see Table 1). Although theoretical, empirical, and practical arguments supporting two-item scales were presented in the “Question Design” section, multi-item scales are more prevalent, and potential measurement limitations of two-item scales may exist. Second, the referent in the items (“SHC consumers”) may not represent a subjectively meaningful or cohesive social group for all respondents. Unlike family or close friends (captured by subjective norms), the generic category “SHC consumers” might lack sufficient identity relevance to exert normative pressure. Third, the null finding could reflect a genuine theoretical absence of group norm effects for this behavior and cohort. Given these possibilities, the nonsignificant result should be interpreted with caution. Future research should employ multi-item scales with more specifically defined referent groups (e.g., “close friends who buy SHC” or “online community members”) to provide a more robust test of this relationship.

Finally, the findings confirm a positive association between social identity and the intention to purchase SHC, which aligns with prior research (Yang, 2019). This effect was medium-to-large in magnitude, suggesting that stronger group identification is meaningfully associated with an increased likelihood of SHC adoption. From a practical standpoint, interventions that support a sense of community and shared identity among SHC consumers may be particularly effective for this demographic.

In conclusion, this study offers a culturally situated extension of the TPB for Chinese Gen Z consumers in the SHC context. Social identity and subjective norm were identified as the most consistent predictors, while the roles of PBC and group norm remained equivocal. Before discussing the implications, it is essential to reiterate the study's methodological constraints. The data are cross-sectional and self-reported, and the sample is non-probability. Consequently, no causal inferences can be drawn from any path coefficient, and the observed associations may not generalize to the broader Chinese Gen Z population. The model comparisons are descriptive and in-sample only; out-of-sample validation is required. These caveats apply to all findings reported below.

### 5.2 Theoretical implications

This study makes several significant theoretical contributions to the literature on SHC consumption. First, it addresses a demographic gap by examining Chinese Gen Z consumers, a cohort situated at the intersection of Confucian collectivism and digital individualism (Tsai and Bagozzi, 2014). Prior research has predominantly focused on young female consumers in developed countries or urban college students in developing nations (Kian et al., 2022; Madeline et al., 2023; Tryphena and Arul Aram, 2023). Initial, exploratory evidence is provided by the present findings regarding which psychological and social factors are associated with SHC purchase intention within this understudied population.

Second, the TPB framework is extended in a culturally situated manner by the present research. Criticisms that the TPB overemphasizes rational self-interest (ElHaffar et al., 2020) were addressed through the incorporation of biospheric values, altruistic values, and three distinct social influence constructs. Comparisons of structural models revealed that, within the current sample, the integrated model explained more variance than the baseline TPB. This improvement suggests that the addition of value-based and social constructs may be useful when sustainable fashion adoption is studied in collectivist, digitally immersed cultures.

Third, three forms of social influence were compared within a single model. The strongest positive correlation with purchase intention was observed for social identity. No significant correlation was found for group norm. This pattern, as observed in the present sample, suggests that for Chinese Gen Z consumers, internalized group identification may be more relevant than perceived pressure from generic reference groups. However, the nonsignificant finding for group norm may also be attributable to measurement limitations (i.e., a two-item scale and a generic referent) rather than to a genuine theoretical boundary. Future research employing more specific referents (e.g., “close friends who buy SHC”) is needed to determine whether group norm effects emerge under improved measurement conditions.

### 5.3 Practical implications

The findings offer several potential implications for both enterprises and governments. First, the positive association observed between consumer attitude and purchase intention suggests that strategic marketing efforts could be directed toward emphasizing the multidimensional value proposition of SHC to support a favorable consumer mindset (Kian et al., 2022). Concurrently, it may be beneficial to address potential consumer concerns, particularly those related to perceived risks associated with second-hand fashion consumption (Kian et al., 2024). However, given the cross-sectional nature of the data, it is equally possible that purchase intention is associated with attitude, therefore, iterative campaigns that both shape and respond to consumer attitudes should be considered by managers.

Second, a significant positive association was observed between subjective norm and purchase intention, indicating that marketing strategies leveraging social networks to amplify positive word-of-mouth could be effective (Tu and Hu, 2018). Given the pervasive role of social media in its association with consumer behavior, SHC brands and government agencies might encourage existing customers and influencers to share favorable experiences and content (Joshi et al., 2025). Such an approach may help support a social environment that is conducive to the adoption of SHC.

Third, associations of biospheric and altruistic values with both attitude and purchase intention were identified, suggesting that promotional campaigns could be designed to incorporate messaging highlighting the societal and ecological benefits of SHC consumption. Such messaging may be associated with a more favorable consumer attitude and a stronger purchase intention.

Fourth, a positive association was observed between social identity and purchase intention (β = 0.394), suggesting that brand managers could benefit from facilitating community-building among SHC consumers. This might be achieved by creating platforms through which like-minded individuals can connect and by implementing collaborative initiatives that reinforce shared values and social identity. Such strategies may prove to be particularly effective in enhancing consumer engagement and loyalty (Yang, 2019). However, because reverse causality cannot be ruled out due to the cross-sectional design, identity-based interventions should be tested using experimental designs prior to full deployment.

Future research employing longitudinal or experimental designs is needed to validate these practical suggestions before they can be adopted as firm guidelines.

## 6 Conclusions, limitations and future research

### 6.1 Conclusions

This study was designed to address a gap in the sustainable consumption literature by examining factors associated with SHC purchase intentions among the influential Chinese Gen Z consumers. Although the TPB offers a foundational model, its application to this specific cultural and generational context may be enhanced through theoretical extension. Accordingly, an integrated framework was developed and empirically tested, incorporating biospheric values, altruistic values, and social influence alongside the core TPB constructs of attitude, PBC, and subjective norm.

Several findings emerged from the analysis. First, the extended model explained a substantially higher proportion of variance in purchase intention (53.7%) compared to the baseline TPB model (34.2%). This suggests that integrating value-based and social constructs may be useful when examining sustainable fashion adoption within a collectivist, digitally immersed culture. However, because the model comparison was based on in-sample fit indices, out-of-sample validation is needed to confirm the generalizability of this explanatory advantage. Second, subjective norm (β = 0.187) showed a slightly stronger positive association with purchase intention than did attitude (β = 0.168). This pattern may reflect the role of social perceptions in being associated with consumption decisions for this cohort, but the difference was small, suggesting caution against overinterpreting the dominance of subjective norm. PBC was not significantly associated with purchase intention. One possible explanation is the high digital fluency of Gen Z consumers, for whom only small barriers to SHC procurement are perceived. Alternative explanations are discussed in the Discussion section. Social identity emerged as the strongest correlate of purchase intention (β = 0.394), indicating that group identification may be particularly relevant for this demographic.

Useful directions for future research and for marketing practitioners intending to pilot interventions are offered by the validated measurement model and the observed correlational patterns. A context-sensitive, extended TPB framework for Chinese Gen Z consumers is offered by this study, thereby contributing to the literature on environmental sustainability, the circular economy, and culturally situated consumer behavior. The utility of the framework for causal explanation and population generalization remains to be established in future research.

### 6.2 Limitations and future research

Several limitations should be considered when interpreting the findings. These limitations also offer directions for future research. First, a nonprobability stratified quota sampling method was employed, based on city tiers (first, second, third, or below). No random selection was applied within strata, nor were the data weighted to match population parameters (e.g., age, gender, and education distributions of Chinese Gen Z). A comparison of sample demographics with the latest census data for Chinese Gen Z (e.g., by gender, education, and urban-rural distribution) was not possible due to lack of accessible population parameters. They represent associational patterns observed within a large but nonrepresentative sample. Future research should adopt probability-based sampling techniques or, at a minimum, provide such comparisons.

Second, all data were collected at a single time point. Future research should therefore employ longitudinal designs or experimental manipulations to test directional hypotheses.

Third, the study relied entirely on self-reported measures, which are susceptible to social desirability bias—particularly for environmentally relevant values and behaviors. Although Harman's single-factor test and the ULMC suggested that common method bias was not pervasive, these tests do not rule out social desirability. Moreover, the outcome variable was purchase intention rather than actual purchase behavior. The well-documented intention-behavior gap (Sheeran and Webb, 2016) implies that the observed correlates of intention may not be directly reflected in SHC purchases. Future research should complement self-reports with behavioral observations (e.g., actual purchase records and digital trace data from resale platforms) or employ indirect questioning techniques to reduce social desirability.

Fourth, two constructs were measured using two-item scales. Although the Spearman-Brown coefficient and factor loadings were acceptable, two-item measures are more susceptible to measurement error and may not fully capture the breadth of the constructs. Additionally, social identity was measured with a pictorial scale, whereas the other constructs were assessed using Likert scales. Even though the ULMC did not detect systematic method effects, different response formats may still introduce unwanted variance. Future research should use multi-item scales with consistent response formats.

Fifth, the null effects for PBC and group norm should not be interpreted as evidence of no relationship. These null findings may reflect ceiling effects (for PBC), low construct relevance (for group norm), or insufficient statistical power to detect small effects. Given the sample size, power was adequate for medium-to-large effects, but small yet meaningful effects could remain undetected. Future research should test these relationships using samples with greater variability in PBC and more specifically defined reference groups (e.g., “close friends who buy SHC” rather than generic “SHC consumers”).

Sixth, although the HTMT value (0.288) confirmed empirical distinctness between biospheric and altruistic values, their path coefficients were nearly identical for both purchase intention and attitude, and their inter-construct correlation was the highest among all nonidentical constructs in the study. This near-parallel pattern raises the possibility that, in our sample, biospheric and altruistic values may not operate as fully separable drivers but rather as a unified self-transcendence orientation. Future research could formally test a higher-order self-transcendence factor that subsumes both value types to determine whether they coalesce into a broader motivational construct in similar behavioral contexts.

## Data Availability

The datasets presented in this article are not readily available because as the data in this study are derived from an ongoing series of research projects that have not yet been finalized, they are subject to the research data management policy of the author's institution (Zhejiang Sci-Tech University) and are therefore not currently suitable for public deposition in an open repository. Requests to access the datasets should be directed to Qi Zhou, 1078655090@qq.com.
